# Effect of Calcium and Vitamin D Supplements as an Adjuvant Therapy to Metformin on Menstrual Cycle Abnormalities, Hormonal Profile, and IGF-1 System in Polycystic Ovary Syndrome Patients: A Randomized, Placebo-Controlled Clinical Trial

**DOI:** 10.1155/2019/9680390

**Published:** 2019-07-01

**Authors:** Sally Kadoura, Marwan Alhalabi, Abdul Hakim Nattouf

**Affiliations:** ^1^Department of Pharmaceutics and Pharmaceutical Technology, Faculty of Pharmacy, Damascus University, Damascus, Syria; ^2^Department of Embryology and Reproductive Medicine, Faculty of Medicine, Damascus University, Damascus, Syria; ^3^Assisted Reproduction Unit, Orient Hospital, Damascus, Syria

## Abstract

**Objective:**

This study aims to investigate the effect of combining calcium and vitamin D supplements with metformin on menstrual cycle abnormalities, gonadotropins, and IGF-1 system in vitamin D-deficient/insufficient PCOS women.

**Study Design:**

This is a randomized, placebo-controlled clinical trial.

**Setting:**

This study was performed in Damascus University of Obstetrics and Gynecology Hospital and Orient Hospital, in Damascus, Syria.

**Materials and Methods:**

Forty PCOS women with 25-OH-vitamin D < 30 ng/ml were randomly assigned to take either metformin (1500 mg/daily) plus placebo or metformin (1500 mg/daily) plus calcium (1000 mg/daily) and vitamin D_3_ (6000 IU/daily) orally for 8 weeks. Serum levels of gonadotropins (luteinizing hormone (LH) and follicle-stimulating hormone (FSH)), insulin-like growth factor-1 (IGF-1), and insulin-like growth factor binding protein-1 (IGFBP-1) were detected at the baseline during the early follicular phase of a spontaneous or induced menstrual cycle and after 8 weeks of intervention (except for the final gonadotropins levels which were assayed from samples obtained during the early follicular phase of a spontaneous menstrual cycle).

**Results:**

Thirty-four patients (85%) completed the study. After 8 weeks of intervention, calcium and vitamin D co-supplementation led to a significant increase in 25-OH-vitamin D levels and calcium levels in the supplementation group compared to the other group (change in 25-OH-vitamin D levels: +19.38 ± 7.78 vs +0.11 ± 4.79 ng/ml, respectively; *p* value=0.0001) (change in calcium levels: +0.83 ± 0.82 vs +0.01 ± 0.86 mg/dl, respectively; *p* value=0.014). An improvement in menstrual cycle irregularity was detected in 38.5% and 58.8% of patients in metformin-placebo group and metformin-calcium-vitamin D group, respectively; but the change was statistically significant only in the supplementation group (*p* value=0.002). Nevertheless, the means of changes from baseline in gonadotropins levels (serum levels of LH, FSH, and LH to FSH ratio) and the studied parameters of IGF-1 system (serum levels of IGF-1, IGFBP-1, and IGF-1 to IGFBP-I ratio) did not differ significantly between the two groups.

**Conclusions:**

Calcium and vitamin D supplements can support metformin effect on regulation of menstrual cycle irregularity in vitamin D-deficient/insufficient PCOS patients, but this effect is not associated with any significant changes in gonadotropins or IGF-1 system. These results suggest a possible role of calcium and vitamin D supplements in managing PCOS. However, further studies are needed to identify the underlying mechanisms. The Clinical Trial Registration Number is NCT03792984.

## 1. Introduction

Polycystic ovary syndrome (PCOS) is the most common endocrine disorder among females of reproductive age, with a worldwide prevalence of 5–20% depending on the criteria used [1, 2]. The main manifestations of this syndrome are ovulatory dysfunction, hyperandrogenism, and polycystic ovarian morphology [2]. Noticeably, PCOS is associated with several metabolic disturbances such as insulin resistance, compensatory hyperinsulinemia, dyslipidemia, and central obesity, which increases the risk for long-term complications such as type 2 diabetes mellitus, metabolic syndrome, and cardiovascular diseases [3]. Moreover, previous data demonstrated that, compared to normo-ovulatory women, PCOS patients might exhibit a dysregulation in the IGF system represented as an elevation in the serum levels of free insulin-like growth factor-1 (IGF-1) and a reduction in the serum levels of insulin-like growth factor binding protein-1 (IGFBP-1) [4]. The IGF system plays an important role in the regulation of oocyte maturation, folliculogenesis, ovarian steroidogenesis, and metabolism [5, 6]. Thus, abnormality in the IGF system may play a role in the pathogenesis of PCOS. IGF-1 is a growth factor with an endocrine action. It is mostly synthesized by the liver but it is also produced by other tissues, including the ovaries, where it has autocrine/paracrine functions [5]. The function and bioavailability of IGF-I are modulated by six binding proteins (IGFBP 1–6) and their proteases [6]. Among the six IGFBPs, IGFBP-1 is the most important binding protein with regard to insulin and glucose metabolism [7]. The IGFBP-1 gene contains an insulin-sensitive response element that directly suppresses its transcription in response to increased hepatic insulin exposure [6]. However, the exact aetiology of PCOS remains unclear and current treatments are only moderately effective at controlling PCOS symptoms and preventing its complications [8]. Thus, seeking for alternative or adjuvant therapies, numerous studies investigated the impact of dietary supplements such as zinc [9], inositol [10], omega-3 fatty acids [11], and vitamins [12] in management of this syndrome. Some of those studies focused on vitamin D. Vitamin D is no longer considered a vitamin solely responsible for regulating bone metabolism and maintaining calcium homeostasis as several studies demonstrated its influence on cell proliferation, differentiation, apoptosis, immune regulation, genome stability, and neurogenesis [13]. Growing evidence suggests a role of vitamin D in female reproductive diseases, as the expression of vitamin D receptors (VDRs) was identified in many organs throughout the female reproductive tract, such as ovary (particularly, granulosa cells), uterus, and placenta [14]. Animal studies revealed that VDR null female mice were infertile and they exhibited uterine hypoplasia and impaired folliculogenesis [15]. On the top of that, vitamin D regulates over 300 genes, including genes that are important for glucose and lipid metabolism. Thus, vitamin D deficiency may be the missing link between insulin resistance and PCOS [3]. Moreover, vitamin D deficiency is a common condition among women with PCOS [16, 17], and several studies indicated an association between low levels of serum 25-hydroxyvitamin D (25-OH-vitamin D) and manifestations of PCOS including insulin resistance [16–20], hyperandrogenism [18], and infertility [21, 22]. Besides, abnormalities in calcium homeostasis and PTH levels secondary to vitamin D deficiency may be responsible for the arrested follicular development and menstrual dysfunction in women with PCOS [23]. Further, a recent in vitro study [24] showed that vitamin D regulated steroidogenesis and IGFBP-1 production in cultured human ovarian cells, and many reports have suggested an interrelation between IGF-1 and vitamin D [25]. However, supplementation of PCOS patients with calcium and vitamin D, with or without metformin, led to divergent outcomes on follicles maturation and menstrual cycle regulation [23, 26–28], and no previous study investigated the effects of calcium or vitamin D supplements on the IGF-1 system in this population. Considering the aforementioned data, we conducted this placebo-controlled clinical trial to assess the effect of calcium and vitamin D supplements as an adjuvant therapy to metformin on menstrual cycle abnormalities, gonadotropins, and IGF-1 system in vitamin D-deficient/insufficient PCOS women.

## 2. Materials and Methods

### 2.1. Study Design

This randomized, single-blinded, placebo-controlled clinical trial was conducted on women with PCOS who referred to the outpatient clinic at Damascus University of Obstetrics and Gynecology Hospital and Orient Hospital, in Damascus, Syria, from December 2016 to December 2017. The Ethical Committee of Damascus University approved the study protocol, and a written informed consent was obtained from all participants. The clinical trial registration number is NCT03792984.

### 2.2. Participants

In this study, we included PCOS women aged 18–30 years. The diagnosis of PCOS was done according to the Rotterdam criteria [29] and the presence of at least two of the following three criteria: (1) oligo or anovulation, (2) clinical and/or biochemical signs of hyperandrogenism, (3) polycystic ovarian morphology on ultrasound examination (defined as the presence of 12 or more follicles in each ovary measuring 2–9 mm in diameter and/or an ovarian volume >10 ml). Patients who were diagnosed with androgen-secreting tumours, Cushing's syndrome, congenital adrenal hyperplasia, hyperprolactinemia, hypercalcemia, malabsorption disorders, diabetes mellitus, thyroid disorders, liver disease, renal disease, history of kidney stones, epilepsy, or cardiovascular disease were excluded. Pregnant, postpartum, or breastfeeding women were excluded as well. All women at the baseline were vitamin D-deficient or insufficient according to the Endocrine Society Clinical Practice Guideline [30]. All study participants reported no use of any hormonal therapy, corticosteroids (other than topical corticosteroids forms), insulin sensitizers, hypolipidemic agents, anti-obesity medications, vitamin D or calcium supplements, anti-epileptic drugs, or any other drugs known to affect endocrine parameters, carbohydrate metabolism, or calciotropic hormone concentrations during the last 3 months. Every patient who met the inclusion criteria and approved to participate in this trial had a face-to-face interview at the baseline to answer a comprehensive questionnaire, which embraced; age, family disease history, smoking habits, alcohol drinking habits, sunscreens usage habits, dress code, and the length of outdoor exposure to the sunlight. All women were advised to maintain their usual dietary habits and not to modify other lifestyle factors such as sunlight exposure or physical activity during the study period.

### 2.3. Intervention, Randomization, and Blindness

Patients were randomly assigned into two study groups when they came back in the early follicular phase (2–5 days of menses) of a spontaneous or induced menstrual cycle. Patients in group A received metformin and placebo; patients in group B received metformin, calcium carbonate, and vitamin D_3_ (cholecalciferol). All treatments were given orally for 8 weeks. The metformin dose was increased stepwise (starting with 500 mg once daily for the 1^st^ week and 500 mg twice daily in the 2^nd^ week, followed by 500 mg 3 times daily from the 3^rd^ week onward). The dose of calcium carbonate (1000 mg/daily) and vitamin D_3_ (6000 IU/daily) remained constant throughout the study period. Simple randomization was done using a randomized table created by computer software (Random Allocation Software Version 1.0.0, Isfahan, Iran). The allocation was not concealed and allocation ratio was 1 : 1. Only the patients were blinded to the identity of the treatment group.

### 2.4. Clinical Assessment

Standard anthropometric data were obtained from each subject (height, weight, waist circumference, and hip circumference), in an overnight fasting status without shoes with light clothes, at the baseline and after 8 weeks of intervention. Body mass index (BMI) was calculated as weight in kilograms divided by height in meters squared (kg/m^2^). Menstrual regularity was assessed as the presence of a menstrual cycle with 21–35 days. Hirsutism was evaluated using the modified Ferriman–Gallwey score (m-FGS) [31], with a threshold (m-FGS) ≥6.

### 2.5. Assessment of Biochemical Variables

All assays were conducted at the laboratories of Damascus University of Obstetrics and Gynecology Hospital. All blood samples were taken after an overnight fast (≈10 hours of fasting). We took 10 millilitres of venous blood from each participant at the baseline during the early follicular phase (2–5 days of menses) of a spontaneous or induced menstrual cycle. After 8 weeks of intervention, other samples were taken to assess the final levels of all biological parameters except for LH and FSH, which were assayed from samples obtained during the early follicular phase of a spontaneous menstrual cycle. Samples were centrifuged at 4000 rpm for 10 min to separate serum. Then, the serum was stored at −60°C until assayed. Serum concentrations of IGF-1 were assayed using an ELISA kit from DIAsource ImmunoAssays S.A. (Belgium) after acid-ethanol extraction. Serum concentrations of IGFBP-1 were assayed using an ELISA kit from DIAsource ImmunoAssays S.A. (Belgium). Serum concentrations of LH, FSH, and 25-OH-vitamin D were assayed using immunofluorescence kits from I-CHROMA (Boditech Med Inc., Korea). Calcium and phosphorus were assayed by the colorimetric method using kits from AMS S.p.A. (Italy). The intra-assay coefficients of variation for all assays were less than 10%. The interassay coefficients of variation for all assays were less than 10% except for IGFBP-1 assay, which was less than 15%.

### 2.6. Statistical Analysis

All statistical analyses were performed using a Statistical Package for the Social Sciences (SPSS) software version 24.0 (IBM Corp., Armonk, NY, USA). Continuous variables were expressed as mean ± standard deviation and categorical variables as counts with percentages. The Kolmogorov–Smirnov test was used to evaluate the normality of data distribution. Between-group comparisons were performed using the independent *t*-test for normally distributed variables, the Mann–Whitney *U* test for nonnormally distributed variables, and chi-square or Fisher's exact test as appropriate for categorical variables. Within-group comparisons were performed using the paired *t*-test for normally distributed variables, the Wilcoxon paired rank test for nonnormally distributed variables, and the McNemar's test for categorical variables. For testing all hypotheses, tests were two-tailed, and *p* values less than 0.05 were considered statistically significant.

## 3. Results

**Figure 1:**
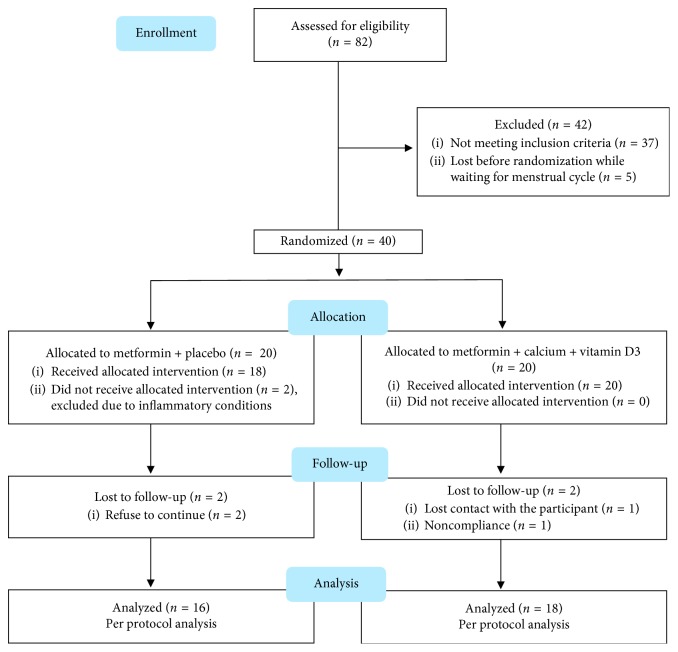
Flow diagram of the study.

Of 82 patients with PCOS, 45 women met the inclusion criteria and participated in the study, of which 5 patients were lost before randomization while waiting for their menses. Forty patients were randomly assigned into two groups, with 20 patients in each group. However, only 34 patients (85%) completed the study (group A: metformin-placebo, *n*=16; group B: metformin-calcium-vitamin D_3_, *n*=18). The details about the study design and subjects lost to follow-up are illustrated in Figure 1. At the baseline, the two groups did not differ significantly in age, BMI, or other baseline characteristics, as shown in Table 1. After 8 weeks of intervention, calcium and vitamin D co-supplementation led to a significant increase in 25-OH-vitamin D levels and calcium levels in the supplementation group compared to the other group (change in 25-OH-vitamin D levels: +19.38 ± 7.78 vs +0.11 ± 4.79 ng/ml, respectively; *p* value=0.0001) (change in calcium levels: +0.83 ± 0.82 vs +0.01 ± 0.86 mg/dl, respectively; *p* value=0.014), but no significant change was detected in phosphorus levels (change in phosphorus levels: +0.38 ± 0.85 vs +0.26 ± 1.78 mg/dl, respectively; *p* value=0.281) (Table 2). Weight, BMI, waist circumference, hip circumference, and waist-to-hip ratio decreased significantly in both groups, but the means of changes from baseline did not differ significantly between them. Compared to baseline, a significant decrease in LH was observed in group A (*p* value=0.028), a significant increase in FSH was observed in group B (*p* value=0.037), and a significant decrease in LH to FSH ratio was observed in both groups (*p* values=0.027 and 0.003 in groups A and B, respectively). Nevertheless, the means of changes from baseline did not differ significantly between the two groups. As shown in Table 3, an improvement in menstrual cycle irregularity was detected in 38.5% and 58.8% of patients in groups A and B, respectively; but the change was statistically significant only in the supplementation group (*p* value=0.002). No significant changes were noticed in IGFBP-1 levels or IGF-1 to IGFBP-1 ratio in both groups. Furthermore, although a significant decrease in IGF-1 levels was detected in the supplementation group, the means of changes from baseline did not differ significantly between the two groups. Notably, the serum levels of 25-OH-vitamin D levels were normalized in all patients of group B after supplementation without reaching the toxic level (100 ng/ml). No serious adverse effects were observed in any participant in both groups. Two patients in the metformin-placebo group (12.50%) had headache compared to 5 patients in the metformin-calcium-vitamin D group (27.78%), and the differences between the two groups were insignificant (*p* value=0.405). Five patients in the metformin-placebo group (31.25%) had gastrointestinal side effects compared to 3 patients in the metformin-calcium-vitamin D group (16.67%), and the differences between the two groups were insignificant (*p* value=0.429). All side effects were tolerable.

## 4. Discussion

The results of our study indicated that adding calcium and vitamin D supplements to metformin led to a superior effect on regulation of menstrual cycles in vitamin D-deficient/insufficient subjects with PCOS with no significant effects on serum levels of LH, FSH, LH to FSH ratio, IGF-1, IGFBP-1, or IGF-1 to IGFBP-1 ratio. Recent data revealed that losing weight might improve menstrual irregularity in PCOS patients [32]. However, it is unlikely that the improvement in anthropometric parameters was the main cause of our results, as the means of changes from baseline did not differ significantly between the two groups, and menstrual cycle irregularity improved significantly only in the supplementation group. In agreement with our findings, the single-arm study of Thys-Jacobs et al. [23] showed that treatment of 13 vitamin D-deficient/insufficient PCOS women with calcium (1500 mg/daily) and vitamin D (ergocalciferol (D_2_) 50,000°IU/weekly or biweekly to attain a targeted serum 25-OH-vitamin D concentration of 30–40 ng/ml) for 2 months normalized menstrual cycle irregularity in 7 patients, led to pregnancy in 2 patients, and maintained the menstrual cycle regularity in the other 4 patients who already had normal menstrual cycles before treatment. Moreover, Tehrani et al.'s study [26] demonstrated that the frequency of regular menstrual cycles and dominant follicles (detected using transabdominal sonography) in vitamin D-deficient PCOS women were higher after treatment with calcium (1000 mg/daily) and vitamin D (50,000 IU/every 2 weeks) supplements in addition to metformin (1500 mg/daily) for 4 months compared to metformin alone, calcium-vitamin D alone, or placebo. However, in this study, both metformin and metformin plus calcium-vitamin D therapies significantly improved menstrual irregularity and follicular maturation with better results in the metformin plus calcium-vitamin D group (45% vs 65% for menstrual irregularity and 50% vs 60% for follicular maturation). On the other hand, Rashidi et al.'s study [27] showed that treating PCOS subjects with metformin (1500 mg/daily) plus calcium (1000 mg/daily) and vitamin D (400 IU/daily) for 3 months led to a significantly higher number of dominant follicles (≥14 mm) during the further 2–3 months of follow-up compared to treatment with metformin alone or calcium-vitamin D alone using transvaginal sonography. Besides, the follicular response was relatively higher in the calcium-vitamin D group compared to the metformin group, but the differences between the groups were statistically insignificant. In addition, the improvement in menstrual irregularity after 3 months of intervention was more noticeable in the metformin plus calcium and vitamin D group, though the differences between the groups were also statistically insignificant. Differently, Firouzabadi et al.'s study [28] showed a better improvement in menstrual irregularity, follicle maturation (detected using transvaginal sonography), and infertility after treating PCOS patients with metformin (1500 mg/daily) plus calcium (1000 mg/daily) and vitamin D (100,000 IU/monthly) for 6 months compared to metformin alone, but these results were statistically insignificant. It is worth mentioning that except for Tehrani et al.'s study, previous studies were lack of blindness or placebo controlling. Considering that the differences between the results could be stemmed from the differences in the study designs, the methods were used to detect follicular maturation, baseline vitamin D status, treatment strategies (dose and duration of vitamin D supplementation), and levels of vitamin D after treatment. In our trial, all participants were vitamin D-deficient or insufficient at the baseline (had baseline 25-OH-vitamin D less than 30 ng/ml), and treatment with vitamin D supplements normalized 25-OH-vitamin D levels in all subjects in the supplementation group. So far, the potential mechanism by which vitamin D influences folliculogenesis is still unclear. In the present study, we could not detect any superior effects on improving serum levels of LH, FSH, or LH to FSH ratio in the supplementation group compared to the other group. This is consistent with the results reported by Selimoglu et al.'s [33] and Irani et al.'s [34] studies. However, some reports have suggested that vitamin D effects on folliculogenesis may be mediated by its impact on anti-Mullerian hormone (AMH), in addition to its effect on regulation of calcium homeostasis as the latter plays an important role in oocyte activation and maturation resulting in the resumption and progression of follicular development [14, 23]. AMH is a glycoprotein produced by granulosa cells of primary, preantral, and small antral follicles [14]. AMH levels rise in women with PCOS and may play a role in the pathophysiology of this syndrome [35], as AMH inhibits the recruitment of primordial follicles, decreases the follicular sensitivity to FSH, and inhibits granulosa cell aromatase, leading to an increase in intrafollicular androgen levels [14, 36]. In vitro studies showed that the human AMH gene promoter contains a functional vitamin D responsive element (VDRE) [37], and treating human cumulus granulosa cells with vitamin D led to a downregulation in AMH receptor-II (AMHR-II) [38]. Furthermore, a recent interventional study revealed that treating PCOS women with vitamin D supplements normalized their serum AMH levels. However, this effect was not observed in the control subjects [36]. On the other hand, Asemi et al.'s study [39] showed an improvement in insulin sensitivity after supplementation of PCOS subjects with calcium and vitamin D supplements. Several published reports confirmed the expression of VDR and 25-hydroxy-vitamin D3-1*α*-hydroxylase in pancreatic islets [40, 41]. Furthermore, vitamin D may activate the transcription of human insulin receptor gene as the promoter of this gene has a vitamin D responsive element (VDRE) [42], besides its effects on regulation of extracellular and intracellular calcium as insulin secretion from *β* cells is a calcium-dependent process [43]. Thus, the beneficial effects of calcium and vitamin D in management of PCOS subjects may be a result of maintaining calcium homeostasis, improving insulin sensitivity, and reducing AMH level. However, further studies are needed to confirm that.

Concerning IGF-1 system, several studies have suggested a bidirectional link between IGF-1 and vitamin D. However, both vitamin D and IGF-I are largely expressed throughout the body and have a broad spectrum of effects so their interrelations are extremely complex [25]. Data from previous in vitro and animal studies demonstrated that IGF-1 could be a regulator of renal 25-OH-vitamin D-1*α*-hydroxylase [44, 45]. On the other hand, VDR knockout mice exhibited reduced levels of plasma IGF-I compared to the wild-type mice [46]. An in vitro study on cultured human fetal epiphyseal chondrocytes disclosed that vitamin D stimulated the expression of IGF-1, IGFBP-3, and growth hormone receptor (GHR) [47]. Some reports hypothesized that vitamin D may influence circulating IGF-1 by targeting the liver, as this organ accounts for most IGF-I in the bloodstream and nonparenchymal hepatic cells (stellate, Kupffer, and sinusoidal endothelial) strongly express the VDR and contribute to the pool of liver-derived circulating IGF-I [25]. However, studies on human subjects ended up with inconsistent outcomes. The study of Hyppönen et al. [48] showed a positive correlation between 25-OH-vitamin D and IGF-I, with a linear increase in IGF-1 until 25-OH-vitamin D concentrations reached 30–34 ng/ml, after which this effect reached a plateau. On the other hand, Bogazzi et al.'s study [49] on healthy subjects demonstrated a positive correlation between 25-OH-vitamin D and IGF-I through all values of vitamin D with serum IGF-I concentrations significantly lower in individuals with severe vitamin D deficiency than those with mild-to-absent deficit. Differently, Lumachi et al. [50] did not notice any correlation between 25-OH-vitamin D and IGF-1 in elderly women, while Trummer et al. [51] showed that, in subjects with arterial hypertension, IGF-1 significantly correlated with 1,25-(OH)_2_-vitamin D but not with 25-OH-vitamin D. On the contrary, Zofková et al. [52], in their study about age-associated bone loss in women, did not notice any associations between IGF-I levels and serum 25-OH-vitamin D or 1,25-(OH)_2_-vitamin D. We have to keep in mind that different commercial IGF-I assay kits can give very different results for the same sample, with up to a 2.5-fold difference between the lowest and highest values. This intermethod variability is generally explained by calibration against different IGF-I reference preparations and differences in the efficiency of methods used to remove IGFBPs [53]. However, since association does not mean causation, intervention studies are necessary to prove the latter. Recently, Al-Daghri et al. [54] showed that treating vitamin D-deficient subjects with vitamin D supplements for 6 months (50,000 IU/weekly for the first 2 months, then twice a month for 2 months, followed by 1000 IU/daily in the last 2 months) led to a significant increase in IGF-1 and IGF-1 to IGFBP-3 ratio. Moreover, Ameri et al. [55] noticed an increase in IGF-1 after treatment with vitamin D supplements with a dose 7000 IU/weekly for 12 weeks compared to a dose 5000 IU/weekly or controls (controls received no intervention), so they suggested that the effect was dose dependent. However, both studies were not blinded or placebo-controlled. Actually, the result of our study was in line with previous randomized placebo-controlled trials. A study on Latino and African American vitamin D-deficient/insufficient prediabetes subjects [56] could not detect any significant effects on serum IGF-1 after treatment with a high dose of vitamin D supplements (85,300 IU ± 16,000) for one year. Neither could Trummer et al. [51] in their short-term (8 weeks) study on vitamin D-deficient/insufficient hypertension patients using 2800 IU/daily of vitamin D. Thus, despite using a higher dose than was used in Ameri et al.'s study, those studies failed to detect any significant effect on IGF-1 in short-term or long-term period in vitamin D-deficient/insufficient subjects. Nevertheless, we have to keep in mind that these results were obtained from subjects who belonged to different study populations with different patient's medical records. Recent data showed that many drugs may affect the IGF system like ACE inhibitors (drugs are used for management hypertension) [57] and statins [58, 59] (drugs are used for management dyslipidemia). Thus, concurrent drug usage may be a confounder affecting the outcomes. However, our subjects were not taken any drugs might affect the IGF-1 system or other hormonal or metabolic parameters except for the studied treatments.

To our best knowledge, this is the first randomized, single-blind, placebo-controlled interventional study that investigates the effect of calcium and vitamin D supplements as an adjuvant therapy to metformin on menstrual cycle abnormalities, gonadotropins, and IGF-1 system in vitamin D-deficient/insufficient PCOS women as the latter issue has never been addressed before. On the other hand, some limitations must be considered in the interpretation of our findings. The main limitations of the present study are the small sample size and the absence of follicular growth evaluation on ultrasound examination at the end of the study. Besides, due to lack of budget, we did not assess the effect of studied treatments on other IGFBPs.

## 5. Conclusions

Calcium and vitamin D can support metformin effect on regulation of menstrual cycle irregularity in vitamin D-deficient/insufficient PCOS patients, but this effect is not associated with any significant changes in gonadotropins or IGF-1 system. These results suggest a possible role of calcium and vitamin D supplements in management of PCOS. However, further studies are needed to identify the underlying mechanisms.

## Figures and Tables

**Table 1:** Baseline characteristics of study subjects in both groups.

Variables	Metformin + placebo (*n*=16)	Metformin + calcium + vitamin D_3_ (*n*=18)	*P* value
Age (years)	23.38 ± 3.54	23.06 ± 3.32	0.788
Weight (kg)	71.39 ± 14.79	64.29 ± 13.02	0.147
Height (cm)	159.29 ± 7.84	158.86 ± 5.59	0.853
BMI (kg/m^2^)	28.01 ± 4.41	25.48 ± 4.97	0.128
Family history of DM, % (*n*)	50.0 (8)	27.8 (5)	0.291
Family history of CVD, % (*n*)	37.5 (6)	61.1 (11)	0.303
Family history of thyroid diseases, % (*n*)	0.0 (0)	16.7 (3)	0.230
Family history of PCOS, % (*n*)	25.0 (4)	27.8 (5)	1.000
Smoking, % (*n*)	50.0 (8)	27.8 (5)	0.291
Drinking alcohol, % (*n*)	6.3 (1)	5.6 (1)	1.000
Menstrual irregularity, % (*n*)	81.3 (13)	94.4 (17)	0.323
Hirsutism, % (*n*)	75.0 (12)	77.8 (14)	1.000
Daily outdoors exposure to the sunlight: >30 minutes, % (*n*)	62.5 (10)	44.4 (8)	0.327
Sunscreen use, % (*n*)	62.5 (10)	61.1 (11)	1.000
Hijab, % (*n*)	75.0 (12)	61.1 (11)	0.477

BMI: body mass index; DM: diabetes mellitus; CVD: cardiovascular diseases; PCOS: polycystic ovary syndrome.

**Table 2:** Clinical and hormonal parameters of study subjects in both groups before and after 8 weeks of intervention.

	Metformin + placebo (*n*=16)			*P* value^*∗*^	Metformin + calcium + vitamin D_3_ (*n*=18)			*P* value^*∗*^	*P* value^#^	*P* value^‡^
	Before	After	Mean change		Before	After	Mean change			
Weight (kg)	71.39 ± 14.79	70.48 ± 15.19	−0.91 ± 1.67	0.047	64.29 ± 13.02	62.54 ± 13.10	−1.76 ± 2.23	0.004	0.147	0.223
BMI (kg/m^2^)	28.01 ± 4.41	27.63 ± 4.35	−0.39 ± 0.66	0.032	25.48 ± 4.97	24.78 ± 4.99	−0.70 ± 0.90	0.004	0.128	0.255
Waist circumference (cm)	90.88 ± 12.66	88.75 ± 13.53	−2.13 ± 2.09	0.007	84.61 ± 10.43	81.58 ± 10.31	−3.03 ± 3.12	0.003	0.124	0.135
Hip circumference (cm)	106.25 ± 9.12	104.94 ± 9.28	−1.31 ± 1.35	0.001	103.50 ± 10.46	101.28 ± 10.57	−2.22 ± 2.13	0.0001	0.423	0.153
Waist/hip	0.85 ± 0.06	0.84 ± 0.07	−0.01 ± 0.02	0.026	0.82 ± 0.04	0.80 ± 0.04	−0.01 ± 0.03	0.039	0.088	0.825
LH (mIU/ml)	10.03 ± 5.55	6.86 ± 3.76	−3.17 ± 5.20	0.028	7.73 ± 5.17	6.10 ± 3.23	−1.63 ± 3.04	0.149	0.144	0.403
FSH (mIU/ml)	6.33 ± 2.10	6.89 ± 1.91	0.56 ± 1.16	0.073	5.45 ± 1.73	6.17 ± 1.67	0.72 ± 1.36	0.037	0.187	0.707
LH/FSH	1.77 ± 1.26	1.02 ± 0.48	−0.74 ± 1.22	0.027	1.45 ± 0.94	1.04 ± 0.68	−0.41 ± 0.51	0.003	0.506	0.721
IGF-1 (ng/ml)	193.61 ± 90.25	184.33 ± 72.39	−9.28 ± 93.01	0.695	208.56 ± 106.52	180.35 ± 86.72	−28.21 ± 46.33	0.019	0.664	0.450
IGFBP-1 (ng/ml)	5.59 ± 5.47	4.71 ± 3.30	−0.88 ± 5.48	0.918	7.42 ± 6.14	7.50 ± 8.73	0.08 ± 6.32	0.879	0.368	0.959
IGF-1/IGFBP-1	89.69 ± 123.30	91.02 ± 126.77	1.33 ± 84.84	0.951	76.01 ± 104.57	43.61 ± 37.54	−32.40 ± 74.49	0.231	0.463	0.670
Vitamin D (ng/ml)	19.88 ± 3.92	19.99 ± 5.78	0.11 ± 4.79	0.928	20.42 ± 6.10	39.80 ± 5.55	19.38 ± 7.78	0.0001	0.764	0.0001
Calcium (mg/dl)	9.40 ± 0.71	9.41 ± 0.66	0.01 ± 0.86	0.977	9.09 ± 0.43	9.92 ± 0.84	0.83 ± 0.82	0.0001	0.126	0.014
Phosphorus (mg/dl)	3.82 ± 0.83	4.08 ± 1.51	0.26 ± 1.78	0.875	3.56 ± 0.72	3.94 ± 0.50	0.38 ± 0.85	0.072	0.330	0.281

BMI: body mass index; LH: luteinizing hormone; FSH: follicle-stimulating hormone; IGF-1: insulin-like growth factor-1; IGFBP-1: insulin-like growth factor binding protein-1; ^*∗*^within-group comparison between baseline and 8 weeks of intervention (pretest vs posttest); ^#^between-group comparison at baseline (pretest metformin-placebo group vs pretest metformin-calcium-vitamin D_3_ group); ^‡^: between-group comparison in means of changes from baseline (posttest minus pretest) (means of changes in metformin-placebo group vs means of changes in metformin-calcium-vitamin D_3_ group).

**Table 3:** Improvement in menstrual irregularity in both groups after intervention.

	Metformin + placebo (*n*=16)	*P* value	Metformin + calcium + vitamin D_3_ (*n*=18)	*P* value
Improvement in menstrual irregularity, % (*n*)	38.5% (5/13)	0.062	58.8% (10/17)	0.002

## Data Availability

The statistical data used to support the findings of this study are included in the article.

## References

[1] Goodarzi M. O., Dumesic D. A., Chazenbalk G., Azziz R. (2011). Polycystic ovary syndrome: etiology, pathogenesis and diagnosis. *Nature Reviews Endocrinology*.

[2] Azziz R., Carmina E., Chen Z. (2016). Polycystic ovary syndrome. *Nature Reviews Disease Primers*.

[3] Krul-Poel Y. H. M., Snackey C., Louwers Y. (2013). The role of vitamin D in metabolic disturbances in polycystic ovary syndrome: a systematic review. *European Journal of Endocrinology*.

[4] van Dessel H. J. H. M. T., Lee P. D. K., Faessen G., Fauser B. C. J. M., Giudice L. C. (1999). Elevated serum levels of free insulin-like growth factor I in polycystic ovary syndrome. *Journal of Clinical Endocrinology & Metabolism*.

[5] De Leo V., Musacchio M. C., Cappelli V. (2016). Genetic, hormonal and metabolic aspects of PCOS: an update. *Reproductive Biology and Endocrinology*.

[6] Clemmons D. R. (2018). 40 YEARS OF IGF1: role of IGF-binding proteins in regulating IGF responses to changes in metabolism. *Journal of Molecular Endocrinology*.

[7] Bae J. H., Song D. K., Im S. S. (2013). Regulation of IGFBP-1 in metabolic diseases. *Journal of Lifestyle Medicine*.

[8] Raja-khan N., Stener-victorin E., Wu X., Legro R. S. (2011). The physiological basis of complementary and alternative medicines for polycystic ovary syndrome. *American Journal of Physiology-Endocrinology and Metabolism*.

[9] Foroozanfard F., Jamilian M., Jafari Z. (2015). Effects of zinc supplementation on markers of insulin resistance and lipid profiles in women with polycystic ovary syndrome: a randomized, double-blind, placebo-controlled trial. *Experimental and Clinical Endocrinology & Diabetes*.

[10] Costantino D., Minozzi G., Minozzi F., Guaraldi C. (2009). Metabolic and hormonal effects of myo-inositol in women with polycystic ovary syndrome: a double-blind trial. *European Review for Medical and Pharmacological Sciences*.

[11] Mohammadi E., Rafraf M., Farzadi L., Asghari-Jafarabadi M., Sabour S. (2012). Effects of omega-3 fatty acids supplementation on serum adiponectin levels and some metabolic risk factors in women with polycystic ovary syndrome. *Asia Pacific Journal of Clinical Nutrition*.

[12] Asemi Z., Karamali M., Esmaillzadeh A. (2014). Metabolic response to folate supplementation in overweight women with polycystic ovary syndrome: a randomized double‐blind placebo‐controlled clinical trial. *Molecular Nutrition & Food Research*.

[13] Wang H., Chen W., Li D. (2017). Vitamin D and chronic diseases. *Aging and Disease*.

[14] Irani M., Merhi Z. (2014). Role of vitamin D in ovarian physiology and its implication in reproduction: a systematic review. *Fertility and Sterility*.

[15] Yoshizawa T., Handa Y., Uematsu Y. (1997). Mice lacking the vitamin D receptor exhibit impaired bone formation, uterine hypoplasia and growth retardation after weaning. *Nature Genetics*.

[16] Wehr E., Pilz S., Schweighofer N. (2009). Association of hypovitaminosis D with metabolic disturbances in polycystic ovary syndrome. *European Journal of Endocrinology*.

[17] Li H. W. R., Brereton R. E., Anderson R. A., Wallace A. M., Ho C. K. M. (2011). Vitamin D deficiency is common and associated with metabolic risk factors in patients with polycystic ovary syndrome. *Metabolism*.

[18] Hahn S., Haselhorst U., Tan S. (2006). Low serum 25-hydroxyvitamin D concentrations are associated with insulin resistance and obesity in women with polycystic ovary syndrome. *Experimental and Clinical Endocrinology & Diabetes*.

[19] Mishra S., Das A. K., Das S. (2016). Hypovitaminosis D and associated cardiometabolic risk in women with PCOS. *Journal of Clinical and Diagnostic Research*.

[20] Patra S. K., Nasrat H., Goswami B., Jain A. (2012). Vitamin D as a predictor of insulin resistance in polycystic ovarian syndrome. *Diabetes & Metabolic Syndrome: Clinical Research & Reviews*.

[21] Pal L., Zhang H., Williams J. (2016). Vitamin D status relates to reproductive outcome in women with polycystic ovary syndrome: secondary analysis of a multicenter randomized controlled trial. *Journal of Clinical Endocrinology & Metabolism*.

[22] Ott J., Wattar L., Kurz C. (2012). Parameters for calcium metabolism in women with polycystic ovary syndrome who undergo clomiphene citrate stimulation: a prospective cohort study. *European Journal of Endocrinology*.

[23] Thys-Jacobs S., Donovan D., Papadopoulos A., Sarrel P., Bilezikian J. P. (1999). Vitamin D and calcium dysregulation in the polycystic ovarian syndrome. *Steroids*.

[24] Parikh G., Varadinova M., Suwandhi P. (2010). Vitamin D regulates steroidogenesis and insulin-like growth factor binding protein-1 (IGFBP-1) production in human ovarian cells. *Hormone and Metabolic Research*.

[25] Ameri P., Giusti A., Boschetti M., Murialdo G., Minuto F., Ferone D. (2013). Interactions between vitamin D and IGF-I: from physiology to clinical practice. *Clinical Endocrinology*.

[26] Tehrani H. G., Mostajeran F., Shahsavari S. (2014). The effect of calcium and vitamin D supplementation on menstrual cycle, body mass index and hyperandrogenism state of women with poly cystic ovarian syndrome. *Journal of Research in Medical Sciences*.

[27] Rashidi B., Haghollahi F., Shariat M., Zayerii F. (2009). The effects of calcium-vitamin D and metformin on polycystic ovary syndrome: a pilot study. *Taiwanese Journal of Obstetrics and Gynecology*.

[28] Firouzabadi R. d., Aflatoonian A., Modarresi S., Sekhavat L., MohammadTaheri S. (2012). Therapeutic effects of calcium & vitamin D supplementation in women with PCOS. *Complementary Therapies in Clinical Practice*.

[29] The Rotterdam ESHRE/ASRM-Sponsored PCOS Consensus Workshop Group (2004). Revised 2003 consensus on diagnostic criteria and long-term health risks related to polycystic ovary syndrome. *Fertility and Sterility*.

[30] Holick M. F., Binkley N. C., Bischoff-Ferrari H. A. (2011). Evaluation, treatment, and prevention of vitamin D deficiency: an endocrine society clinical practice guideline. *Journal of Clinical Endocrinology & Metabolism*.

[31] Yildiz B. O., Bolour S., Woods K., Moore A., Azziz R. (2010). Visually scoring hirsutism. *Human Reproduction Update*.

[32] Marzouk T. M., Sayed Ahmed W. A. (2015). Effect of dietary weight loss on menstrual regularity in obese young adult women with polycystic ovary syndrome. *Journal of Pediatric and Adolescent Gynecology*.

[33] Selimoglu H., Duran C., Kiyici S. (2010). The effect of vitamin D replacement therapy on insulin resistance and androgen levels in women with polycystic ovary syndrome. *Journal of Endocrinological Investigation*.

[34] Irani M., Seifer D. B., Grazi R. V. (2015). Vitamin D supplementation decreases TGF-*β*1 bioavailability in PCOS: a randomized placebo-controlled trial. *Journal of Clinical Endocrinology & Metabolism*.

[35] Garg D., Tal R. (2016). The role of AMH in the pathophysiology of polycystic ovarian syndrome. *Reproductive BioMedicine Online*.

[36] Irani M., Minkoff H., Seifer D. B., Merhi Z. (2014). Vitamin D increases serum levels of the soluble receptor for advanced glycation end products in women with PCOS. *Journal of Clinical Endocrinology & Metabolism*.

[37] Malloy P. J., Peng L., Wang J., Feldman D. (2009). Interaction of the vitamin D receptor with a vitamin D response element in the Müllerian-inhibiting substance (MIS) promoter: regulation of MIS expression by calcitriol in prostate cancer cells. *Endocrinology*.

[38] Merhi Z., Doswell A., Krebs K., Cipolla M. (2014). Vitamin D alters genes involved in follicular development and steroidogenesis in human cumulus granulosa cells. *Journal of Clinical Endocrinology & Metabolism*.

[39] Asemi Z., Foroozanfard F., Hashemi T., Bahmani F., Jamilian M., Esmaillzadeh A. (2015). Calcium plus vitamin D supplementation affects glucose metabolism and lipid concentrations in overweight and obese vitamin D deficient women with polycystic ovary syndrome. *Clinical Nutrition*.

[40] Bland R., Markovic D., Hills C. E. (2004). Expression of 25-hydroxyvitamin D3-1*α*-hydroxylase in pancreatic islets. *Journal of Steroid Biochemistry and Molecular Biology*.

[41] Wang Y., Zhu J., DeLuca H. F. (2012). Where is the vitamin D receptor?. *Archives of Biochemistry and Biophysics*.

[42] Maestro B., Dávila N., Carranza M. C., Calle C. (2003). Identification of a vitamin D response element in the human insulin receptor gene promoter. *Journal of Steroid Biochemistry and Molecular Biology*.

[43] Sung C.-C., Liao M.-T., Lu K.-C., Wu C.-C. (2012). Role of vitamin D in insulin resistance. *Journal of Biomedicine and Biotechnology*.

[44] Nesbitt T., Drezner M. K. (1993). Insulin-like growth factor-I regulation of renal 25-hydroxyvitamin D-1- hydroxylase activity. *Endocrinology*.

[45] Menaa C., Vrtovsnik F., Friedlander G., Corvol M., Garabedian M. (1995). Insulin-like growth factor I, a unique calcium-dependent stimulator of 1,25-dihydroxyvitamin D3 production: Studies in cultured mouse kidney cells. *Journal of Biological Chemistry*.

[46] Song Y., Kato S., Fleet J. C. (2003). Vitamin D receptor (VDR) knockout mice reveal VDR-independent regulation of intestinal calcium absorption and ECaC2 and calbindin D9k mRNA. *Journal of Nutrition*.

[47] Fernández-Cancio M., Audi L., Carrascosa A. (2009). Vitamin D and growth hormone regulate growth hormone/insulin-like growth factor (GH-IGF) axis gene expression in human fetal epiphyseal chondrocytes. *Growth Hormone & IGF Research*.

[48] Hypponen E., Boucher B. J., Berry D. J., Power C. (2008). 25-hydroxyvitamin D, IGF-1, and metabolic syndrome at 45 years of age: a cross-sectional study in the 1958 British Birth Cohort. *Diabetes*.

[49] Bogazzi F., Rossi G., Lombardi  M. (2011). Vitamin D status may contribute to serum insulin-like growth factor I concentrations in healthy subjects. *Journal of Endocrinological Investigation*.

[50] Lumachi F., Camozzi V., Doretto P., Tozzoli R., Basso S. M. M. (2013). Circulating PTH, vitamin D and IGF-I levels in relation to bone mineral density in elderly women. *In Vivo*.

[51] Trummer C., Schwetz V., Pandis M. (2017). Effects of vitamin D supplementation on IGF-1 and calcitriol: a randomized-controlled trial. *Nutrients*.

[52] Zofková I., Bahbouh R., Bendlová B. (2001). Systemic insulin-like growth factor-I, insulin and vitamin D status in relation to age-associated bone loss in women. *Experimental and Clinical Endocrinology & Diabetes*.

[53] Chanson P., Arnoux A., Mavromati M. (2016). Reference values for IGF-I serum concentrations: comparison of six immunoassays. *Journal of Clinical Endocrinology & Metabolism*.

[54] Al-Daghri N. M., Yakout S. M., Wani K. (2018). IGF and IGFBP as an index for discrimination between vitamin D supplementation responders and nonresponders in overweight Saudi subjects. *Medicine*.

[55] Ameri P., Giusti A., Boschetti M. (2013). Vitamin D increases circulating IGF1 in adults: potential implication for the treatment of GH deficiency. *European Journal of Endocrinology*.

[56] Sinha-Hikim I., Duran P., Shen R. (2015). Effect of long term vitamin d supplementation on biomarkers of inflammation in Latino and African-American subjects with pre-diabetes and hypovitaminosis D. *Hormone and Metabolic Research*.

[57] Giovannini S., Cesari M., Marzetti E., Leeuwenburgh C., Maggio M., Pahor M. (2010). Effects of ACE-inhibition on IGF-1 and IGFBP-3 concentrations in older adults with high cardiovascular risk profile. *Journal of Nutrition, Health & Aging*.

[58] Bergen K., Brismar K., Tehrani S. (2016). High-dose atorvastatin is associated with lower IGF-1 levels in patients with type 1 diabetes. *Growth Hormone & IGF Research*.

[59] Narayanan R. P., Gittins M., Siddals K. W. (2013). Atorvastatin administration is associated with dose-related changes in IGF bioavailability. *European Journal of Endocrinology*.

